# In-silico characterization of the relationship between the Porcine reproductive and respiratory syndrome virus prevalence at the piglet and litter levels in a farrowing room

**DOI:** 10.1186/s40813-023-00309-x

**Published:** 2023-04-13

**Authors:** Onyekachukwu H. Osemeke, Eduardo de Freitas Costa, Vinicius Weide, Swaminathan Jayaraman, Gustavo S. Silva, Daniel C. L. Linhares

**Affiliations:** 1 Veterinary Diagnostic and Production Animal Medicine, College of Veterinary Medicine, Iowa State University, 2422 Lloyd, 1809 S Riverside Dr, Ames, IA 50011-3619 USA; 2 Department of Epidemiology, Bioinformatics, and Animal Models, Wageningen Bioveterinary Research, Lelystad, The Netherlands; 3 Instituto Federal de Educação, Ciência e Tecnologia do Rio Grande do Sul, Farroupilha, RS Brazil

**Keywords:** Prevalence, Simulations, FOF, Clustering, PRRSV, Piglets

## Abstract

**Background:**

Family oral fluids (FOF) sampling has been described as a sampling technique where a rope is exposed to sows and respective suckling litters and thereafter wrung to obtain fluids. PCR-based testing of FOF reveals presence of PRRS virus RNA only at the litter level, as opposed to conventional individual-animal-based sampling methods that demonstrate PRRSV RNA at the piglet level. The relationship between the PRRSV prevalence at the individual piglet level and at the litter level in a farrowing room has not been previously characterized. Using Monte Carlo simulations and data from a previous study, the relationship between the proportion of PRRSV-positive (viremic) pigs in the farrowing room, the proportion of litters in the farrowing room with at least one viremic pig, and the likely proportion of litters to be positive by a FOF RT-rtPCR test in a farrowing room was characterized, taking into account the spatial distribution (homogeneity) of viremic pigs within farrowing rooms.

**Results:**

There was a linear relationship between piglet-level- and litter-level prevalence, where the latter was always larger than the former. When the piglet-level prevalence was 1%, 5%, 10%, 20%, and 50%, the true-litter level prevalence was 5.36%, 8.93%, 14.29%, 23.21%, and 53.57%, respectively. The corresponding apparent-litter prevalence by FOF was 2.06%, 6.48%, 11.25%, 21.60%, and 51.56%, respectively.

**Conclusion:**

This study provides matching prevalence estimates to help guide sample size calculations. It also provides a framework to estimate the likely proportion of viremic pigs, given the PRRSV RT-rtPCR positivity rate of FOF samples submitted from a farrowing room.

**Supplementary Information:**

The online version contains supplementary material available at 10.1186/s40813-023-00309-x.

## Background

Porcine reproductive and respiratory syndrome virus (PRRSV) poses a significant challenge to the swine industry causing spikes in mortality rates, abortion rates, feed conversion ratios, time to market, and costs of medication; it was estimated to cost the US swine industry over 600 million USD annually [1, 2].

Monitoring and surveillance systems remains an integral component of PRRSV control and elimination programs; and ascertaining the PRRSV status of weaning-age pig populations is crucial to guide decisions on health interventions and pig flow [3]. Efficient PRRSV surveillance/monitoring programs allow for the early detection of infection and helps evaluate changes in PRRSV prevalence over time; aiding swine producers and veterinarians alike to forestall the spread of PRRSV [4, 5], and evaluate progress made with instituted PRRSV management programs [6, 7].

Different sample types are routinely submitted to veterinary diagnostic laboratories in the United States for PRRSV reverse transcription real time polymerase chain reaction (RT-rtPCR) tests; these would include samples taken from individual pigs such as serum, swabs, semen, and post-mortem tissues; or aggregate samples taken from multiple pigs such as processing fluids and oral fluids [8]. These samples are either submitted and tested individually or in pools.

The number of samples submitted for pathogen investigation is crucial to the success of a surveillance/monitoring exercise. Guided by epidemiological and statistical assumptions, the sample size should have enough power to detect at least one positive unit if the herd is truly positive for the pathogen of interest [9, 10].

Estimated prevalence at the individual pig level is one of the key variables used in calculating sample size to demonstrate the presence of a pathogen in a herd [9, 11]. The diagnostic sample of choice for PRRSV surveillance in sow herds is serum from weaning-age pigs [3]. Although serum is the sample of choice, it requires more skill, more manpower, is less animal welfare friendly, and is often impractical for frequent PRRSV surveillance in large herds [12] compared to population-based sampling options. For these reasons, since 2018, aggregated samples have been the most frequently submitted samples for PRRSV surveillance in the US [8].

Almeida et al. [13] demonstrated that, especially at low prevalence, FOF sampling is a more convenient and cost-efficient alternative to serum sampling for PRRSV detection in weaning-age pigs. A FOF sample is an aggregate sample obtained when oral fluids are wrung off a rope chewed by a sow and her suckling piglets [13]. A challenge with interpreting a positive result from FOF and other aggregate sample types is the uncertainty on the number of pigs that contributed to the sample matrix, or the number of PRRSV-positive animals if the sample tests RT-rtPCR positive for PRRSV RNA.

The individual pig is the unit for which sample size is calculated when non-aggregate samples are collected, while the litter is the unit for which sample size is calculated when an aggregate sample such as FOF is to be collected [14, 15]. To make more accurate sampling decision, swine practitioners need to understand how the proportion of PRRSV-positive (viremic) piglets relates with the proportion of PRRSV-positive litters, as both parameters are needed assumptions in estimating sample sizes. The relationship between the piglet-level- and litter-level prevalence in swine farrowing rooms has not been previously characterized in available literature.

Therefore, the objective of this in-silico study was to characterize the relationship between the piglet-level prevalence (PP), true litter-level PRRSV prevalence (TLP) and apparent litter-level PRRSV prevalence (ALP) in a farrowing room.

## Results

### PRRSV detection in pig litters using FOF

**Fig. 1:**
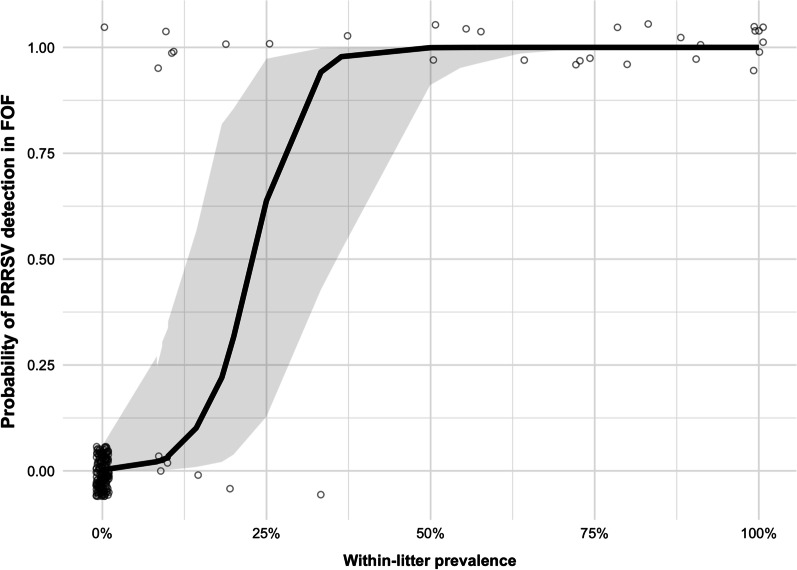
A jitter plot of the probability of PRRSV RNA detection in FOF by the proportion of positive pigs within litters (within litter prevalence). The 95% prediction intervals are represented by the grey region around the regression line

The probability of PRRSV RNA detection in FOF by RT-rtPCR increased with increasing proportion of PRRSV-positive piglets within a litter. There was a 95% probability of PRRSV detection in FOF when within-litter prevalence was 35% or higher (Fig. 1, Additional file 1: Table S2). The AUC of the predictive model was 0.9915 (Additional file 1: Fig. S1).

### Stochastic model

#### Observed distribution of clustering in sampled farms

**Fig. 2:**
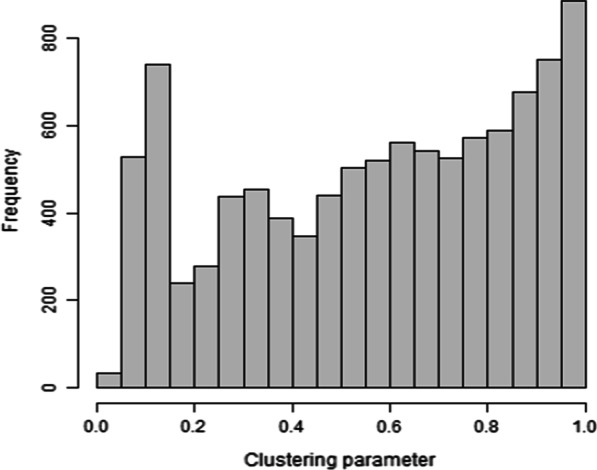
Distribution of the clustering parameter $\hat{c}$ across all sampled rooms

The clustering distribution across all sampled rooms had a minimum value of 0.00136, a median of 0.61000, a mean of 0.57000, and a maximum value of 1.0000. The distributions of the clustering parameter across all sampled rooms are presented in Fig. 2.

#### The relationship between piglet-level prevalence and litter-level prevalence

**Fig. 3:**
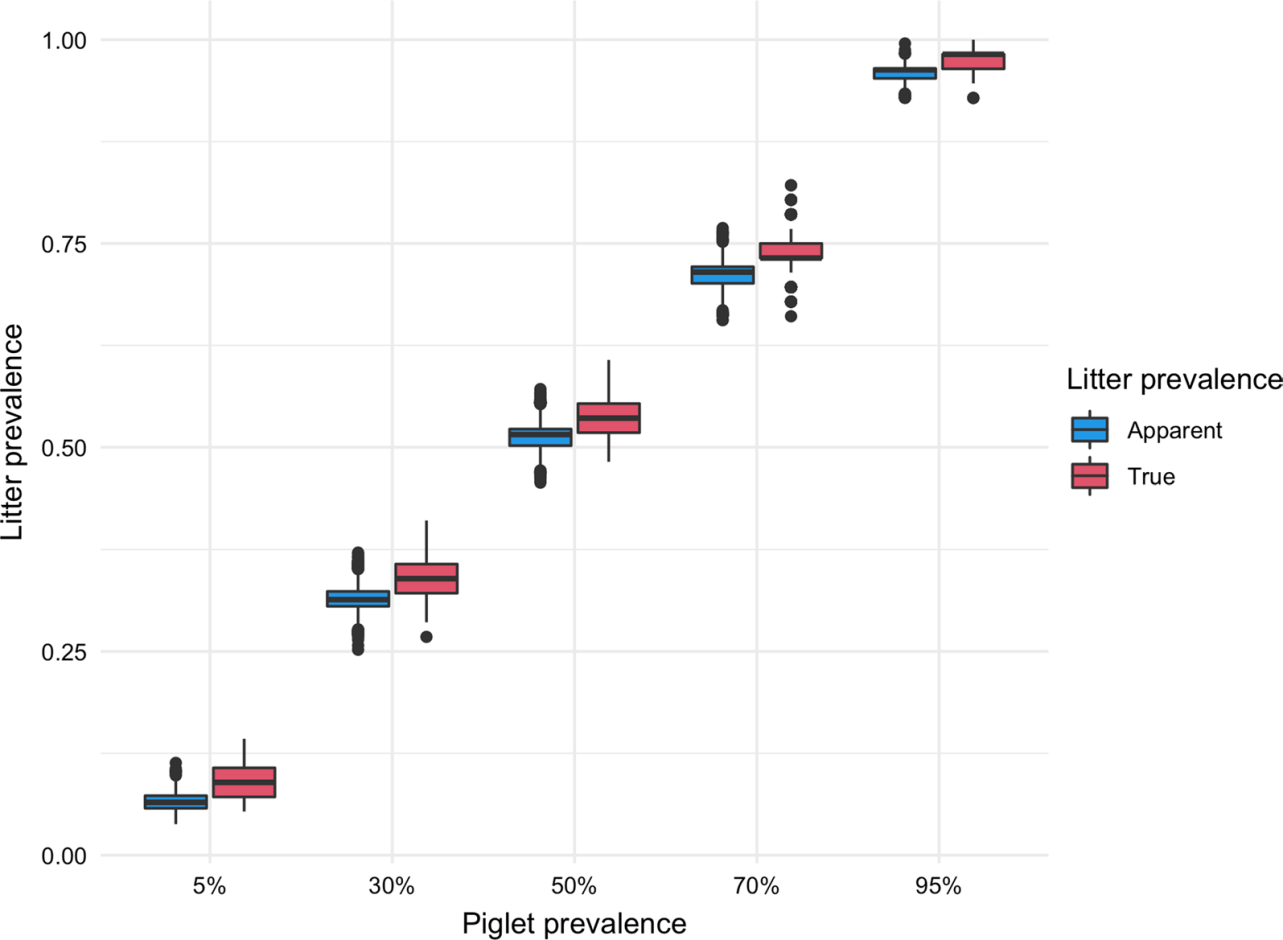
Distribution of true- and apparent litter prevalence in a 56-crate room given different piglet-level prevalence scenarios and a clustering factor of 0.61

Table 1 and Fig. 3 show changes in median TLP and median ALP with increasing proportion of PRRSV-positive pigs in a 56-crate farrowing room considering a clustering factor of 0.61. When 1% of the piglets in the room are PRRSV-positive, about 5.36% of the 56 crates (~ 3 crates) are expected to have at least 1 PRRSV-positive piglet, and 2.06% of the 56 crates (~ 1 crate) is expected to give a positive FOF.

**Table 1:** Relationship between the proportion of positive piglets in a 56-crate farrowing room and the true and apparent (by FOF) proportion of positive litters assuming a clustering level of 0.61

Proportion of PRRSV-positive individual piglets (%)	True proportion of PRRSV-positive litters (upper and lower 95% quantiles) (%)	Apparent proportion of PRRSV-positive litters by FOF (upper and lower 95% quantiles) (%)
1	5.36 (1.79, 7.14)	2.06 (1.07, 3.53)
5	8.93 (7.14, 12.50)	6.48 (5.30, 8.58)
10	14.29 (10.71, 17.86)	11.25 (9.31, 13.92)
15	19.64 (16.07, 23.21)	16.35 (14.47, 19.21)
20	23.21 (21.43, 26.79)	21.60 (18.73, 24.19)
25	28.57 (25.00, 32.14)	26.66 (23.50, 29.31)
30	33.93 (30.36, 37.50)	31.35 (28.77, 34.33)
35	39.29 (35.71, 42.86)	36.16 (33.49, 39.44)
40	44.64 (41.07, 48.21)	41.30 (38.05, 44.71)
45	48.21 (44.64, 53.57)	46.54 (43.10, 49.68)
50	53.57 (50.00, 57.14)	51.56 (48.34, 54.58)

### Sensitivity analysis

**Fig. 4:**
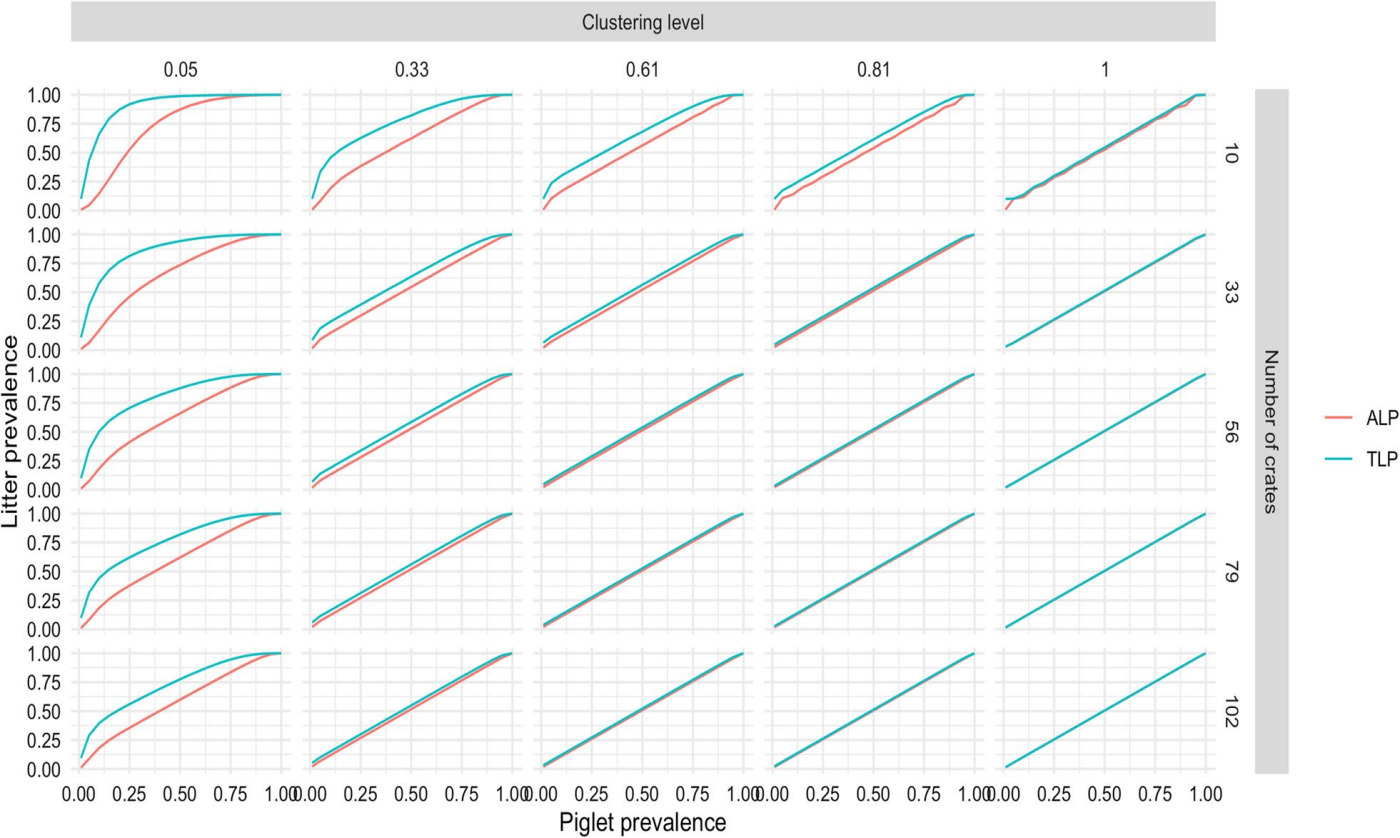
Graphical representation of changes in the relationship between the proportion of PRRSV-positive pigs and the proportion of PRRSV-positive litters (True and Apparent) with changes in clustering of PRRSV within room, and number of litters within rooms

A sensitivity analysis was done to evaluate the effect of variations in clustering level and room size on the proposed relationship between piglet level prevalence and litter prevalence. The ALP was relatively more stable to changes in clustering and the number of crates compared to TLP. Generally, TLP and ALP increasingly converged to similar values with increasing clustering and increasing room size. Clustering changes appeared to have a more significant effect on ALP and TLP than changes in the number of crates in the room (Fig. 4).

## Discussion

This study used mathematical models built upon earlier studies to characterize the relationship between piglet- and litter level PRRSV prevalence in a farrowing room. The use of mathematical models to describe disease dynamics in swine populations is not new. A few examples include the use of mathematical models to characterize and describe PRRSV transmission dynamics [14, 16–20] and in the evaluation of PRRSV control strategies [21, 22].

Earlier studies described the non-homogenous distribution of PRRSV in pig barns [14, 23]; the non-homogenous areal distribution of infectious pathogens however is not limited to PRRSV alone [24, 25]. This phenomenon may be explained by PRRSV being highly infectious but not necessarily highly contagious [26], or by the mere fact that pigs in conventional barns do not interact randomly with each other and are more likely to have direct contact with pigs within the same crate or with their closest neighbors [27].

Some popular statistical methods used in veterinary epidemiology for detecting and evaluating spatial (areal) clustering include Moran’s* I*, ohno, black-white, Geary’s *c*, and *I* pop [24], however, the use of the recursive binomial model in this study offered the authors a method to not only measure clustering, but to also propagate clustering in simulated data. The use of binomial models to detect and simulate clustering is also not new [28, 29].

The restricted movement of pigs in conventional swine barns and the non-homogenous distribution of viremic animals have been historically recognized to make conventional sample size assumptions (to detect a disease pathogen) not an exact fit; some previously proposed solutions include replacing simple random sampling with fixed spatial sampling [14], risk-based sampling [23], or stratified sampling [30]. This study provides another method to adjust conventional sampling schemes to better fit peculiarities with typical modern swine barns and with the ecology of PRRSV.

Clustering estimates the degree of homogeneity (or, more aptly, heterogeneity) of PRRSV between litters in a farrowing room. It may be overreaching to deterministically model a one-size-fits-all clustering for PRRSV because the spread of PRRSV between litters within a farrowing room depends on a variety of factors including: (1) management practices such as cross-fostering, and vaccination [4, 26], (2) PRRSV strain [26, 31–33], (3) barn structure [14], (4) time since outbreak [14], (5) secondary infections which may increase pig susceptibility to PRRSV, encourage huddling or increase the production of infectious respiratory fluids.

The uncertainty in definitively ascertaining clustering level however does not undermine the importance of these results or pose a challenge to its utilization, on the contrary, considering/estimating clustering adds some precision to the estimated prevalence guiding sample size calculations for disease pathogen surveillance (An example is given in Additional file 1: Table S3).

The main goal of this study was to characterize the relationship between the piglet level prevalence and apparent litter prevalence by FOF, considering the pen-level sensitivity and specificity of this sample type. As observed from Fig. 3, ALP is not as sensitive as TLP to variations in clustering parameter. One can also decide the number of crates or litters to randomly sample for FOF to detect PRRSV given an assumed piglet level prevalence. For example, assuming at least 10% piglet-level prevalence, serum sampling requires that about 30 pigs are sampled to be 95% confident of detecting at least one positive animal [3, 34]. From the table, 10% pig-level prevalence corresponds to about 11% ALP or about 7 litters in a 56-crate room likely to give a positive FOF test. This number can be used to calculate an appropriate sample size for FOF to detect at least 1 positive litter; Table 1 is, therefore, useful in estimating the litter prevalence from an assumed piglet-level prevalence.

The approach used in calculating ALP implicitly considers the diagnostic performance of FOF sampling; simply put, for a given piglet-level prevalence, the difference between the ALP and TLP is due to the diagnostic performance of FOF (the probability of RT-rtPCR testing of FOF samples to correctly assign PRRSV statuses to each tested litter). This implies that ALP can be used directly to estimate FOF sample size and the only diagnostic performance that may need to be considered is that of the RT-rtPCR test kit. The AUC (0.9915) of the regression model that characterized the probability of PRRSV RNA detection in FOF by RT-qPCR based on the proportion of viremic pigs within a litter supports that the predictive model performs excellently.

Another key application of the proposed tables is to help the swine practitioner estimate piglet-level prevalence given the results of FOF testing. Given that a representative number of litters were sampled (sample size to estimate prevalence), the proportion of positive FOF results on RT-qPCR tests (apparent litter prevalence by FOF) can be used to deduce the likely proportion of viremic pigs (piglet-level prevalence). In Additional file 1: Table S3, there were scenarios where the ALP was greater than the TLP, for example, the 100% clustering scenarios in the 56 and 102 crate rooms. This is because from the reference study, FOF from one of the sampled litters tested PRRSV-positive by Rt-rtPCR when there was no PRRSV-positive piglet (WLP = 0); consequently, in the predictive model used for the stochastic simulations, the probability of a positive FOF given that WLP is 0, is greater than 0 (Additional file 1: Table S2). A 100% clustering $\left( {c = 1} \right)$ in the stochastic model restricts the distribution of PRRSV-positive pigs to the fewest number of litters possible (hereafter called PRRSV-positive litters), with a consequent maximization of the number of litters without any PRRSV-positive piglets (hereafter called PRRSV-negative litters). The probability of PRRSV-detection in FOF samples obtained from these PRRSV positive litters is almost always 100% owing to relatively high number of PRRSV-positive piglets “concentrated" within each of these litters. This should ordinarily put TLP and ALP at par, but ALP is further increased by the probability of positive FOF RT-rtPCR results from the relatively large number of PRRSV-negative litters. The RT-rtPCR detection of PRRSV RNA in FOF from a PRRSV-negative litter may be as a result of an imperfect test specificity or may be explained by the dam of the litter being PRRSV-positive and shedding (WLP does not consider the PRRSV status of the sow).

The referenced studies [23, 30] were not specifically designed to measure the spatial distribution of viremic piglets within farrowing rooms, as such, in some sampled rooms, not every litter was sampled; consequently, the observed clustering values for those rooms may be inaccurate. To be able to deduce the number of viremic piglets from FOF positivity rate using the provided tables, it is important that one should have sampled the minimum number of litters needed to estimate prevalence.

## Conclusion

This study explored the use of mathematical models to characterize the relationship between PP, TLP, and ALP in a farrowing room.

When the piglet-level prevalence was 1%, 5%, 10%, 20%, and 50%, the true-litter level prevalence was 5.36%, 8.93%, 14.29%, 23.21%, and 53.57%, respectively. The corresponding apparent-litter prevalence by FOF was 2.06%, 6.48%, 11.25%, 21.60%, and 51.56%, respectively.

Prevalence comparisons provided here are intended to help guide sample choice and sample size for PRRSV surveillance in weaning-age pigs. The likely proportion of viremic pigs can also be estimated from the PRRSV-RT-rtPCR positivity rate of tested FOF samples obtained from a farrowing room, using the tables provided.

Further similar studies on other aggregate sample types, for other subpopulations and perhaps, for other pathogens will be helpful in guiding practitioners on how they can be up-to-speed with best practice surveillance as sampling methods evolve.

## Methods

### Overview

A predictive model was first built using data from Almeida et al. [23], to characterize the relationship between within-litter prevalence (the proportion of viremic piglets within a litter) and the probability of a PRRSV-positive FOF sample from that litter. The degrees of clustering (spatial distribution) of PRRSV-positive pigs within sampled rooms from a related study [30] study was measured. An empirical distribution of litter sizes from this reference study was also obtained.

Farrowing rooms were thereafter simulated with a fixed number of litters; the number of pigs within each litter was obtained from the earlier mentioned discrete empirical distribution. A clustering factor was used to distribute viremic pigs between litters, with values ranging from 0 (randomly distributed PRRSV-positive pigs) to 1 (clustering of PRRSV-positive pigs within the fewest litters possible), the baseline clustering was obtained from the reference study. The True litter prevalence per iterated room was obtained as the proportion of litters with at least one viremic pig, and the apparent litter prevalence was obtained as the predicted proportion of the litters in an iterated room that will be PRRSV-positive by FOF testing. A total of 5000 rooms were iterated, and the median values of TLP and ALP were obtained.

### PRRSV detection in pig litters using FOF

Based on a dataset from Almeida et al. [23] 199 litters had all piglets sampled for PRRSV RNA detection by RT-rtPCR (reverse transcription real-time polymerase chain reaction); each litter (*i* = 1,…,199) was also sampled for FOF. The litters were sampled from 11 farrowing rooms across six different swine breeding farms (*j* = 1,…,6).

The effect of the proportion of PRRSV-viremic piglets ($x$) in a litter on the detection of a positive litter using FOF ($P^{FOF}$) was assessed with a generalized linear mixed model employing a logit link function and a 'residual' Bernoulli distribution (i.e., logistic regression). In addition, the linear predictor comprised random effects for farms according to:

1 $$logit\left( {P_{ij}^{FOF} } \right) = \alpha + \beta x_{ij} + \varepsilon_{ij} + \gamma_{j} ,$$

where $\alpha$ is the intercept of the model, $\varepsilon_{i}$ is the random error assumed $\varepsilon_{ij} \sim N\left( {0,\sigma_{\varepsilon } } \right)$, and $\gamma_{j}$ is the random effect accounting for the farm-effect in the model, assumed $\gamma_{j} \sim N\left( {0,\sigma_{\gamma } } \right)$, where all $\varepsilon_{i } and \gamma_{j}$ are independent. Approximate maximum likelihood inference was based upon Laplacian integration, as implemented in R [35] in routine *glmer* from library *lme4* [36]. The numerical value of the area under the receiver operating characteristic curve (AUC) of the model was assessed using the *pROC* package [37] in R [35].

### Stochastic model

The number of RT-rtPCR-positive piglets in the *i-*th litter $\left( {N_{i} } \right)$ is considered a random variable, and assuming that each piglet's status (positive/negative) is a Bernoulli trial, with a fixed *p* probability, $N_{i}$ arises from a binomial process. Consider a room with *n* litters with different sizes $\left( {T_{i} } \right)$ drawn from a discrete empirical distribution, and total number of piglets in the room $T = \mathop \sum \limits_{i = 1}^{n} T_{i}$. In a simplistic scenario, the allocation of positive piglets in each litter $\left( {N_{i} } \right)$ would follow the relative size of the litter in the room. However, when modeling the distribution of pathogens it is expected that the positive animals are not randomly distributed in the room; instead they are clustered in a few litters [24, 25].

Accounting for this, the number of PCR-positive animals in each *i* litter $\left( {N_{i} } \right)$ is calculated as a special case of the multinomial distribution, sampling recursively from binomial distributions using a clustering factor:

2 $$N_{i} [N_{j} \left( {j = 1, \ldots ,i - 1} \right)]\sim min \left\{ {Bin\left[ {\left( {N - \mathop \sum \limits_{j = 1}^{i - 1} N_{j} , pl_{i} } \right)} \right],{\text{T}}_{{\text{i}}} } \right\},$$

where *j* stands for the successive allocation of positive animals within each litter, and $pl$ is the probability of success in this binomial process defined as:

3 $$pl_{i} = \frac{{{\text{T}}_{{\text{i}}} }}{{T - \mathop \sum \nolimits_{j = 1}^{i - 1} T_{j} }} + \left( {1 - \frac{{{\text{T}}_{{\text{i}}} }}{{T - \mathop \sum \nolimits_{j = 1}^{i - 1} T_{j} }}} \right) \cdot c,\;c \in \left[ {0,1} \right].$$

The notation *c* represents a clustering factor. Thus when $\mathop {\lim }\nolimits_{c \to 1} pl = 1$, the positive piglets will be totally clustered in the smallest number of litters as possible. On the other hand, when $\mathop {\lim }\nolimits_{c \to 0} pl = \frac{{{\text{T}}_{{\text{i}}} }}{{T - \mathop \sum \nolimits_{j = 1}^{i - 1} T_{j} }}$, piglets will be spread according to the relative size (number of piglets) of each litter with respect to the room size.

To obtain the baseline clustering factor *c*, the observed distribution of the within litter prevalence $\left( {\theta_{i} } \right)$ reported in Almeida et al. [30] across seven rooms, each room with *n* litters was used. For a farrowing room to be eligible for this study, such room would have ≥ 1 viremic pig, and the litters within these rooms would have been sampled for FOF. Consequently, rooms 1 – 4 of Farm A, rooms 1 and 3 of Farm C, and room 1 of Farm E met the eligibility criteria and were used for this study. There were 89 litters across all seven rooms that were fully sampled for serum and FOF. As would be observed in “Stochastic model” section of the referenced study, a few crates within these rooms were either empty or not sampled.

The lost function was the minimization of the mean squared errors of the predicted (Eq. 2) *vs* observed distribution of the within litter prevalence $f\left( {c,\theta } \right) = \frac{1}{n}\sum\nolimits_{i = 1}^{n} {\left( {\theta_{i} - \frac{{N_{i} }}{{T_{i} }}} \right)^{2} }$. The objective function $f\left( {c,\theta } \right)$ can be used to calculate a parameter estimate $\hat{c} = argmin\left( {f\left( {c,\theta } \right)} \right)$. Each room was randomly chosen 10,000 times obtaining the parameters $\theta_{i}$, $N_{i}$, and $T_{i}$. For each sampled room, 1000 acquisition points in the parameter space of *c* were sampled from a uniform distribution $c\sim uniform\left( {0, 1} \right)$, obtaining a distribution to optimize $\hat{c}$*.*

#### Apparent prevalence at the litter level

The simulated proportions of positive piglets per litter obtained from “Stochastic model” section were used as input for the logistic model fit in “PRRSV detection in pig litters using FOF” section, calculating the probability of detection of each simulated litter using FOF sampling. A random variable (*S*) was modeled describing the most probable number of positive litters detected in a routine FOF sampling in a farrowing room. Assuming the probability of each litter being detected by FOF ($P_{i}^{FOF}$ see Eq. 1) are independent of each other, and the positive/negative status of a litter $y_{i} \sim Bernoulli\left( {P_{i}^{FOF} } \right)$, *S* equals $\sum\nolimits_{i = 1}^{k} {y_{i} }$. The expected apparent litter prevalence $\left( {ALP} \right)$ was obtained as $S/n$. $S$ was generated a total of 2000 times to improve the accuracy of the Monte Carlo estimation, and the mean $ALP$ was obtained and stored for that iterated room.

The parameters and distributions used in the simulations are described in Table 2. In this simulation, 5000 stochastic iterations were performed, each one representing a different room, propagating the between litter variability observable in different farrowing rooms.

**Table 2:** Descriptions of baseline model parameters used to compare the true and apparent liter prevalence of PRRSV

Parameter/variable	Distribution/function	Description	Source
$p$	Fixed = (range of values from 1 to 100%)	Probability of a piglet being positive in a room (prevalence)	Authors’ opinion
$N$	$p \cdot T$	Total number of positive piglets in the room	Calculation
$T$	$\mathop \sum \limits_{i = 1}^{n} T_{i}$	Total number of piglets in the room	Calculation
$T_{i}$	empirical {(), ()}*	Number of piglets in the *i-*th litter	Almeida et al. [23, 30]
$n$	Fixed = 56	Number of crates or litters in a room	Authors’ opinion
$N_{i}$	$min \left\{ {Bin\left[ {\left( {N - \mathop \sum \limits_{j = 1}^{i - 1} N_{j} , pl} \right)} \right],{\text{T}}_{{\text{i}}} } \right\}$	Number of positive piglets in *i-*th litter	Calculation
$pl_{i}$	$\frac{{{\text{T}}_{{\text{i}}} }}{{T - \mathop \sum \nolimits_{j = 1}^{i - 1} T_{j} }} + \left( {1 - \frac{{{\text{T}}_{{\text{i}}} }}{{T - \mathop \sum \nolimits_{j = 1}^{i - 1} T_{j} }}} \right) \cdot c$	Probability of success in this binomial process (i.e., allocation of positive piglets in a litter) for the *i-*th litter	Calculation
$c$	Fixed = 0.61	Clustering factor	Optimized based on Almeida et al. [30]

*Empirical {(3, 4, 5, 6, 7, 8, 9, 10, 11, 12, 13, 14, 15, 25), (0.0092, 0.0092, 0.0046, 0.0046, 0.0553, 0.0691, 0.0922, 0.1014, 0.1982, 0.2074, 0.1244, 0.0783, 0.0415, 0.0046)}

### Sensitivity analysis

To assess the effect of the clustering factor (*c*) and the room size (*n*) on the estimated relationship between pig-level-prevalence and litter-level prevalence, five values for *c* (0.05, 0.34, 0.61, 0.81, 1) and five values for *n* (10, 33, 56, 79, 102) were selected and combined in a factorial design for the sensitivity analysis, totaling 25 different scenarios. The minimum and maximum values for clustering and room sizes were conveniently chosen by the authors, the mid values are the baseline values; and the second and fourth values are calculated midpoints between the baseline and minimum value, and between the baseline and the maximum values respectively.

## Supplementary Information

**Additional file 1. Table S1.** A general description of the stochastic model used for this study, with pictorial illustrations. **Table S2.** The changes in the probability of PRRSV RNA RT-rtPCR detection in FOFs with increases in the proportion of PRRSV viremic piglets within a litter. **Table S3.** The relationship between PP, ALP, and TLP at different clustering levels and room sizes. **Figure S1.** The receiver operating characteristic curve assessing the predictive performance of the model built from Eq. 1.

## Data Availability

The simulations used to generate the figures and tables provided in this manuscript are available in the repository: https://github.com/onyechux/Prevalence-simulations.
