# Supplementary material for: In-silico characterization of the relationship between the Porcine reproductive and respiratory syndrome virus prevalence at the piglet and litter levels in a farrowing room
Source: Porcine Health Manag. 2023 Apr 13;9:14. doi: 10.1186/s40813-023-00309-x (PMC10099699; doi:10.1186/s40813-023-00309-x)
Supplement: Supplementary file 1 — Additional file 1. Table S1. A general description of the stochastic model used for this study, with pictorial illustrations. Table S2. The changes in the probability of PRRSV RNA RT-rtPCR detection in FOFs with increases in the proportion of PRRSV viremic piglets within a litter. Table S3. The relationship between PP, ALP, and TLP at different clustering levels and room sizes. Figure S1. The receiver operating characteristic curve assessing the predictive performance of the model built from Eq. 1. [file 40813_2023_309_MOESM1_ESM.docx]

**SUPPLEMENTARY MATERIALS**

*Table S1: A general description of the stochastic model with pictorial illustrations*


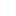


|  | **Goal** | **Example** | **Example in pictures** |
| --- | --- | --- | --- |
| 1 | **To create a farrowing room with *n* litters** by generating *n* random numbers from a discrete empirical distribution corresponding to the average litter size we observed from actual field data | From this empirical distribution, a possible set of random numbers generated will be 8, 9, 9, 7, 9, 8. These numbers are shown in the picture on the right. | 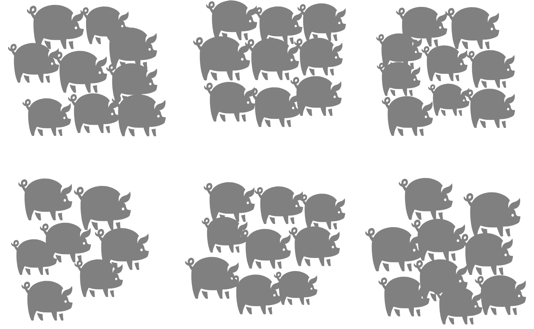 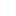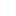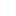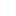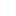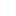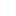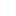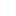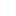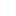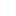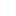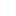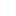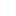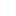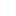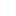 |
| 2. | **To create a disease prevalence scenario within this created room**.  Suppose we wanted a 10% prevalence. We will want 10% of the total pigs in the simulated room to be positive. | When we add all the litters (generated numbers), we have 50 pigs (8+9+9+7+9+8 = 50).  10% of 50 is 5, meaning five pigs will be PRRSV positive | 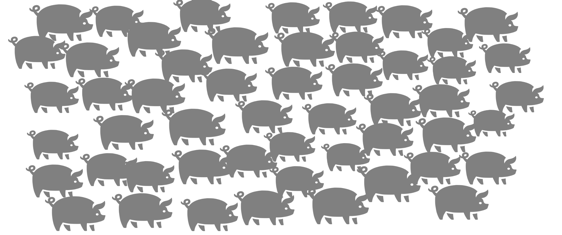  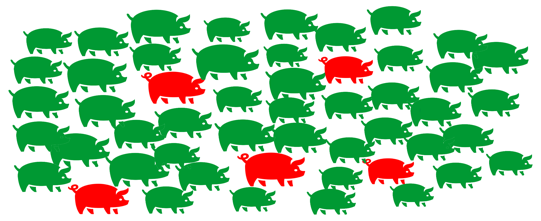 |
| 3 | **To distribute the diseased animals between pens in a manner typical of PRRSV**. PRRSV has been repeatedly reported to be heterogeneously distributed (clustered) within a farrowing room. A clustering factor in the recursive binomial model, which could range between 0 (homogenous distribution) and 1 (complete clustering), was used to assign positive pigs to litters.  The clustering factor determines the True Litter Prevalence (TLP), defined as the number of litters with at least one positive pig. | The first image shows what would be obtainable if there was no clustering (Clustering = 0).  **TLP** = 5/6  The next image shows what is expected in complete clustering (clustering = 1).  **TLP** = 1/6. | 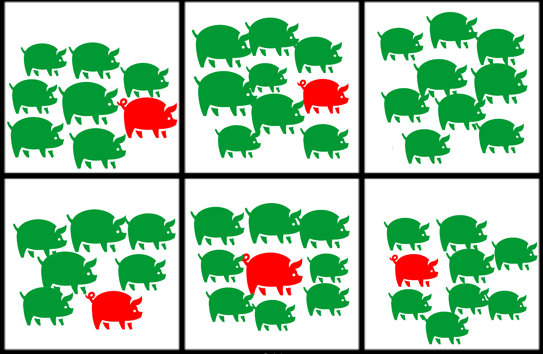  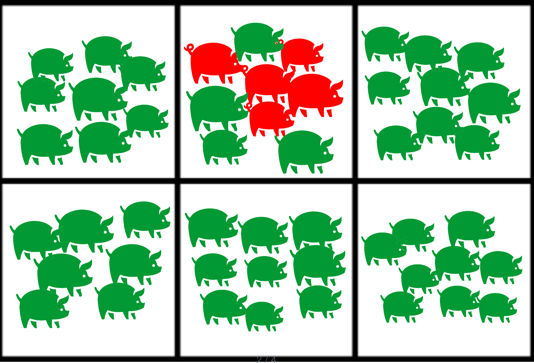 |
| 4 | **To determine the expected number of positive FOF from this room if all the litters are tested**.  A predictive model is fitted using data from a previous study (Almeida, Zhang, Lopez et al., 2021). This model gives the probability of a positive FOF (pFOF) test given the proportion of positive pigs within a litter or within-litter prevalence (WLP).  After these probabilities (pFOFs) are generated, the Expected number of positive FOF tests for that room is determined using a Monte Carlo process. The Apparent litter prevalence by FOF (ALP) is then the expected number of positive FOF divided by the total number of litters or tests | The graph shows the relationship between pFOF and  WLP.  For the clustering = 0, the WLP and pFOF for each litter are calculated.  The expected number of positive FOF for that room is 0.2998 (<1).  **ALP** = <1/6  For the clustering = 1.  The expected number of positive FOF for that room is 1.014 (approximately 1).  **ALP** = 1/6 |    \| WLP = 0.125  pFOF = 0.062 \| WLP = 0.111  pFOF = 0.043 \| WLP = 0  pFOF = 0.002 \| \| --- \| --- \| --- \| \| WLP = 0.143  pFOF = 0.099 \| WLP = 0.111  pFOF = 0.043 \| WLP = 0.111  pFOF = 0.043 \|  \| WLP = 0  pFOF = 0.002 \| WLP = 0.556  pFOF = 1.000 \| WLP = 0  pFOF = 0.002 \| \| --- \| --- \| --- \| \| WLP = 0  pFOF = 0.002 \| WLP = 0  pFOF = 0.002 \| WLP = 0  pFOF = 0.002 \| |
| 5  6 | **Repeat steps 1 to 4 (4,999 more times)** and obtain the median TLP and ALP thereafter. The 5% prevalence is then matched with the median TLP and median ALP for the chosen clustering level.  Then repeat steps 1 to 5 for other prevalence scenarios |  |  |

*Table S2: The changes in the probability of PRRSV RNA RT-rtPCR detection in FOFs with increases in the proportion of PRRSV viremic piglets within a litter*

| **SN** | **Proportion of PRRSV-positive pigs (WLP)** | **Probability of PRRSV RNA RT-rtPCR detection in FOF.** |
| --- | --- | --- |
| 1 | 0.00% | 0.22% |
| 2 | 1.00% | 0.29% |
| 3 | 5.00% | 0.86% |
| 4 | 10.00% | 3.26% |
| 5 | 15.00% | 11.59% |
| 6 | 20.00% | 33.76% |
| 7 | 25.00% | 66.45% |
| 8 | 30.00% | 88.50% |
| 9 | 35.00% | 96.77% |
| 10 | 40.00% | 99.15% |
| 11 | 45.00% | 99.78% |
| 12 | 50.00% | 99.94% |
| 13 | 55.00% | 99.99% |
| 14 | 60.00% | 100.00% |
| 15 | 65.00% | 100.00% |
| 16 | 70.00% | 100.00% |
| 17 | 75.00% | 100.00% |
| 18 | 80.00% | 100.00% |
| 19 | 85.00% | 100.00% |
| 20 | 90.00% | 100.00% |
| 21 | 95.00% | 100.00% |
| 22 | 100.00% | 100.00% |

*Table S3: Relationship between PP, ALP, and TLP at different clustering levels and room sizes*

| **PP**  **(%)** | **Number of crates** | **Clustering**  **(%)** | **TLP**  **(%)** | **ALP**  **(%)** | **Number of litters with at least one PRRSV-positive piglet** | | **Number of litters expected to test PRRSV-positive by FOF sampling** |
| --- | --- | --- | --- | --- | --- | --- | --- |
| 1.00 | 10 | 5.00 | 10.00 | 0.43 | 1 | 0 | |
| 5.00 | 10 | 5.00 | 40.00 | 3.55 | 4 | 0 | |
| 10.00 | 10 | 5.00 | 70.00 | 14.24 | 7 | 1 | |
| 15.00 | 10 | 5.00 | 80.00 | 27.20 | 8 | 3 | |
| 20.00 | 10 | 5.00 | 90.00 | 40.68 | 9 | 4 | |
| 25.00 | 10 | 5.00 | 90.00 | 52.83 | 9 | 5 | |
| 30.00 | 10 | 5.00 | 100.00 | 63.14 | 10 | 6 | |
| 35.00 | 10 | 5.00 | 100.00 | 71.21 | 10 | 7 | |
| 40.00 | 10 | 5.00 | 100.00 | 78.01 | 10 | 8 | |
| 45.00 | 10 | 5.00 | 100.00 | 83.62 | 10 | 8 | |
| 50.00 | 10 | 5.00 | 100.00 | 87.88 | 10 | 9 | |
| 1.00 | 10 | 33.00 | 10.00 | 0.45 | 1 | 0 | |
| 5.00 | 10 | 33.00 | 30.00 | 8.48 | 3 | 1 | |
| 10.00 | 10 | 33.00 | 50.00 | 19.49 | 5 | 2 | |
| 15.00 | 10 | 33.00 | 50.00 | 27.50 | 5 | 3 | |
| 20.00 | 10 | 33.00 | 60.00 | 32.27 | 6 | 3 | |
| 25.00 | 10 | 33.00 | 60.00 | 38.56 | 6 | 4 | |
| 30.00 | 10 | 33.00 | 70.00 | 42.51 | 7 | 4 | |
| 35.00 | 10 | 33.00 | 70.00 | 48.40 | 7 | 5 | |
| 40.00 | 10 | 33.00 | 70.00 | 52.21 | 7 | 5 | |
| 45.00 | 10 | 33.00 | 80.00 | 58.21 | 8 | 6 | |
| 50.00 | 10 | 33.00 | 80.00 | 61.85 | 8 | 6 | |
| 1.00 | 10 | 61.00 | 10.00 | 0.45 | 1 | 0 | |
| 5.00 | 10 | 61.00 | 20.00 | 10.31 | 2 | 1 | |
| 10.00 | 10 | 61.00 | 30.00 | 17.35 | 3 | 2 | |
| 15.00 | 10 | 61.00 | 30.00 | 20.87 | 3 | 2 | |
| 20.00 | 10 | 61.00 | 40.00 | 27.25 | 4 | 3 | |
| 25.00 | 10 | 61.00 | 40.00 | 30.81 | 4 | 3 | |
| 30.00 | 10 | 61.00 | 50.00 | 37.26 | 5 | 4 | |
| 35.00 | 10 | 61.00 | 50.00 | 40.65 | 5 | 4 | |
| 40.00 | 10 | 61.00 | 60.00 | 46.87 | 6 | 5 | |
| 45.00 | 10 | 61.00 | 60.00 | 50.57 | 6 | 5 | |
| 50.00 | 10 | 61.00 | 70.00 | 56.75 | 7 | 6 | |
| 1.00 | 10 | 100.00 | 10.00 | 0.46 | 1 | 0 | |
| 5.00 | 10 | 100.00 | 10.00 | 10.19 | 1 | 1 | |
| 10.00 | 10 | 100.00 | 10.00 | 10.23 | 1 | 1 | |
| 15.00 | 10 | 100.00 | 20.00 | 20.15 | 2 | 2 | |
| 20.00 | 10 | 100.00 | 20.00 | 20.22 | 2 | 2 | |
| 25.00 | 10 | 100.00 | 30.00 | 30.13 | 3 | 3 | |
| 30.00 | 10 | 100.00 | 30.00 | 30.19 | 3 | 3 | |
| 35.00 | 10 | 100.00 | 40.00 | 40.11 | 4 | 4 | |
| 40.00 | 10 | 100.00 | 40.00 | 40.17 | 4 | 4 | |
| 45.00 | 10 | 100.00 | 50.00 | 50.09 | 5 | 5 | |
| 50.00 | 10 | 100.00 | 50.00 | 50.14 | 5 | 5 | |
| 1.00 | 56 | 5.00 | 10.71 | 0.64 | 6 | 0 | |
| 5.00 | 56 | 5.00 | 35.71 | 7.35 | 20 | 4 | |
| 10.00 | 56 | 5.00 | 50.00 | 18.28 | 28 | 10 | |
| 15.00 | 56 | 5.00 | 58.93 | 27.65 | 33 | 15 | |
| 20.00 | 56 | 5.00 | 66.07 | 34.97 | 37 | 20 | |
| 25.00 | 56 | 5.00 | 71.43 | 41.26 | 40 | 23 | |
| 30.00 | 56 | 5.00 | 75.00 | 46.64 | 42 | 26 | |
| 35.00 | 56 | 5.00 | 78.57 | 51.69 | 44 | 29 | |
| 40.00 | 56 | 5.00 | 82.14 | 56.51 | 46 | 32 | |
| 45.00 | 56 | 5.00 | 85.71 | 61.27 | 48 | 34 | |
| 50.00 | 56 | 5.00 | 87.50 | 66.01 | 49 | 37 | |
| 1.00 | 56 | 33.00 | 7.14 | 1.75 | 4 | 1 | |
| 5.00 | 56 | 33.00 | 14.29 | 7.92 | 8 | 4 | |
| 10.00 | 56 | 33.00 | 19.64 | 13.02 | 11 | 7 | |
| 15.00 | 56 | 33.00 | 23.21 | 18.04 | 13 | 10 | |
| 20.00 | 56 | 33.00 | 28.57 | 23.13 | 16 | 13 | |
| 25.00 | 56 | 33.00 | 33.93 | 27.93 | 19 | 16 | |
| 30.00 | 56 | 33.00 | 39.29 | 32.86 | 22 | 18 | |
| 35.00 | 56 | 33.00 | 42.86 | 37.92 | 24 | 21 | |
| 40.00 | 56 | 33.00 | 48.21 | 42.97 | 27 | 24 | |
| 45.00 | 56 | 33.00 | 53.57 | 47.89 | 30 | 27 | |
| 50.00 | 56 | 33.00 | 58.93 | 52.86 | 33 | 30 | |
| 1.00 | 56 | 61.00 | 5.36 | 2.06 | 3 | 1 | |
| 5.00 | 56 | 61.00 | 8.93 | 6.45 | 5 | 4 | |
| 10.00 | 56 | 61.00 | 14.29 | 11.27 | 8 | 6 | |
| 15.00 | 56 | 61.00 | 19.64 | 16.34 | 11 | 9 | |
| 20.00 | 56 | 61.00 | 23.21 | 21.61 | 13 | 12 | |
| 25.00 | 56 | 61.00 | 28.57 | 26.62 | 16 | 15 | |
| 30.00 | 56 | 61.00 | 33.93 | 31.36 | 19 | 18 | |
| 35.00 | 56 | 61.00 | 39.29 | 36.18 | 22 | 20 | |
| 40.00 | 56 | 61.00 | 44.64 | 41.28 | 25 | 23 | |
| 45.00 | 56 | 61.00 | 48.21 | 46.54 | 27 | 26 | |
| 50.00 | 56 | 61.00 | 53.57 | 51.62 | 30 | 29 | |
| 1.00 | 56 | 100.00 | 1.79 | 2.00 | 1 | 1 | |
| 5.00 | 56 | 100.00 | 5.36 | 5.57 | 3 | 3 | |
| 10.00 | 56 | 100.00 | 10.71 | 10.91 | 6 | 6 | |
| 15.00 | 56 | 100.00 | 16.07 | 16.23 | 9 | 9 | |
| 20.00 | 56 | 100.00 | 21.43 | 20.42 | 12 | 11 | |
| 25.00 | 56 | 100.00 | 25.00 | 25.18 | 14 | 14 | |
| 30.00 | 56 | 100.00 | 30.36 | 30.52 | 17 | 17 | |
| 35.00 | 56 | 100.00 | 35.71 | 35.85 | 20 | 20 | |
| 40.00 | 56 | 100.00 | 41.07 | 41.18 | 23 | 23 | |
| 45.00 | 56 | 100.00 | 46.43 | 45.19 | 26 | 25 | |
| 50.00 | 56 | 100.00 | 50.00 | 50.13 | 28 | 28 | |
| 1.00 | 102 | 5.00 | 9.80 | 0.91 | 10 | 1 | |
| 5.00 | 102 | 5.00 | 29.41 | 8.75 | 30 | 9 | |
| 10.00 | 102 | 5.00 | 39.22 | 18.14 | 40 | 19 | |
| 15.00 | 102 | 5.00 | 46.08 | 24.96 | 47 | 25 | |
| 20.00 | 102 | 5.00 | 50.98 | 30.44 | 52 | 31 | |
| 25.00 | 102 | 5.00 | 55.88 | 35.47 | 57 | 36 | |
| 30.00 | 102 | 5.00 | 60.78 | 40.36 | 62 | 41 | |
| 35.00 | 102 | 5.00 | 64.71 | 45.31 | 66 | 46 | |
| 40.00 | 102 | 5.00 | 68.63 | 50.15 | 70 | 51 | |
| 45.00 | 102 | 5.00 | 73.53 | 54.99 | 75 | 56 | |
| 50.00 | 102 | 5.00 | 77.45 | 59.81 | 79 | 61 | |
| 1.00 | 102 | 33.00 | 4.90 | 2.07 | 5 | 2 | |
| 5.00 | 102 | 33.00 | 9.80 | 6.85 | 10 | 7 | |
| 10.00 | 102 | 33.00 | 14.71 | 11.85 | 15 | 12 | |
| 15.00 | 102 | 33.00 | 19.61 | 16.82 | 20 | 17 | |
| 20.00 | 102 | 33.00 | 24.51 | 21.76 | 25 | 22 | |
| 25.00 | 102 | 33.00 | 30.39 | 26.75 | 31 | 27 | |
| 30.00 | 102 | 33.00 | 35.29 | 31.67 | 36 | 32 | |
| 35.00 | 102 | 33.00 | 40.20 | 36.66 | 41 | 37 | |
| 40.00 | 102 | 33.00 | 45.10 | 41.68 | 46 | 43 | |
| 45.00 | 102 | 33.00 | 50.00 | 46.69 | 51 | 48 | |
| 50.00 | 102 | 33.00 | 54.90 | 51.68 | 56 | 53 | |
| 1.00 | 102 | 61.00 | 2.94 | 1.99 | 3 | 2 | |
| 5.00 | 102 | 61.00 | 6.86 | 6.04 | 7 | 6 | |
| 10.00 | 102 | 61.00 | 11.76 | 10.99 | 12 | 11 | |
| 15.00 | 102 | 61.00 | 17.65 | 15.90 | 18 | 16 | |
| 20.00 | 102 | 61.00 | 22.55 | 20.82 | 23 | 21 | |
| 25.00 | 102 | 61.00 | 27.45 | 25.77 | 28 | 26 | |
| 30.00 | 102 | 61.00 | 32.35 | 30.74 | 33 | 31 | |
| 35.00 | 102 | 61.00 | 37.25 | 35.70 | 38 | 36 | |
| 40.00 | 102 | 61.00 | 42.16 | 40.81 | 43 | 42 | |
| 45.00 | 102 | 61.00 | 47.06 | 45.89 | 48 | 47 | |
| 50.00 | 102 | 61.00 | 51.96 | 50.88 | 53 | 52 | |
| 1.00 | 102 | 100.00 | 0.98 | 1.21 | 1 | 1 | |
| 5.00 | 102 | 100.00 | 5.88 | 5.14 | 6 | 5 | |
| 10.00 | 102 | 100.00 | 10.78 | 10.31 | 11 | 11 | |
| 15.00 | 102 | 100.00 | 15.69 | 15.68 | 16 | 16 | |
| 20.00 | 102 | 100.00 | 20.59 | 20.74 | 21 | 21 | |
| 25.00 | 102 | 100.00 | 25.49 | 25.65 | 26 | 26 | |
| 30.00 | 102 | 100.00 | 30.39 | 30.54 | 31 | 31 | |
| 35.00 | 102 | 100.00 | 35.29 | 35.44 | 36 | 36 | |
| 40.00 | 102 | 100.00 | 40.20 | 40.33 | 41 | 41 | |
| 45.00 | 102 | 100.00 | 45.10 | 45.23 | 46 | 46 | |
| 50.00 | 102 | 100.00 | 50.00 | 50.12 | 51 | 51 | |

*
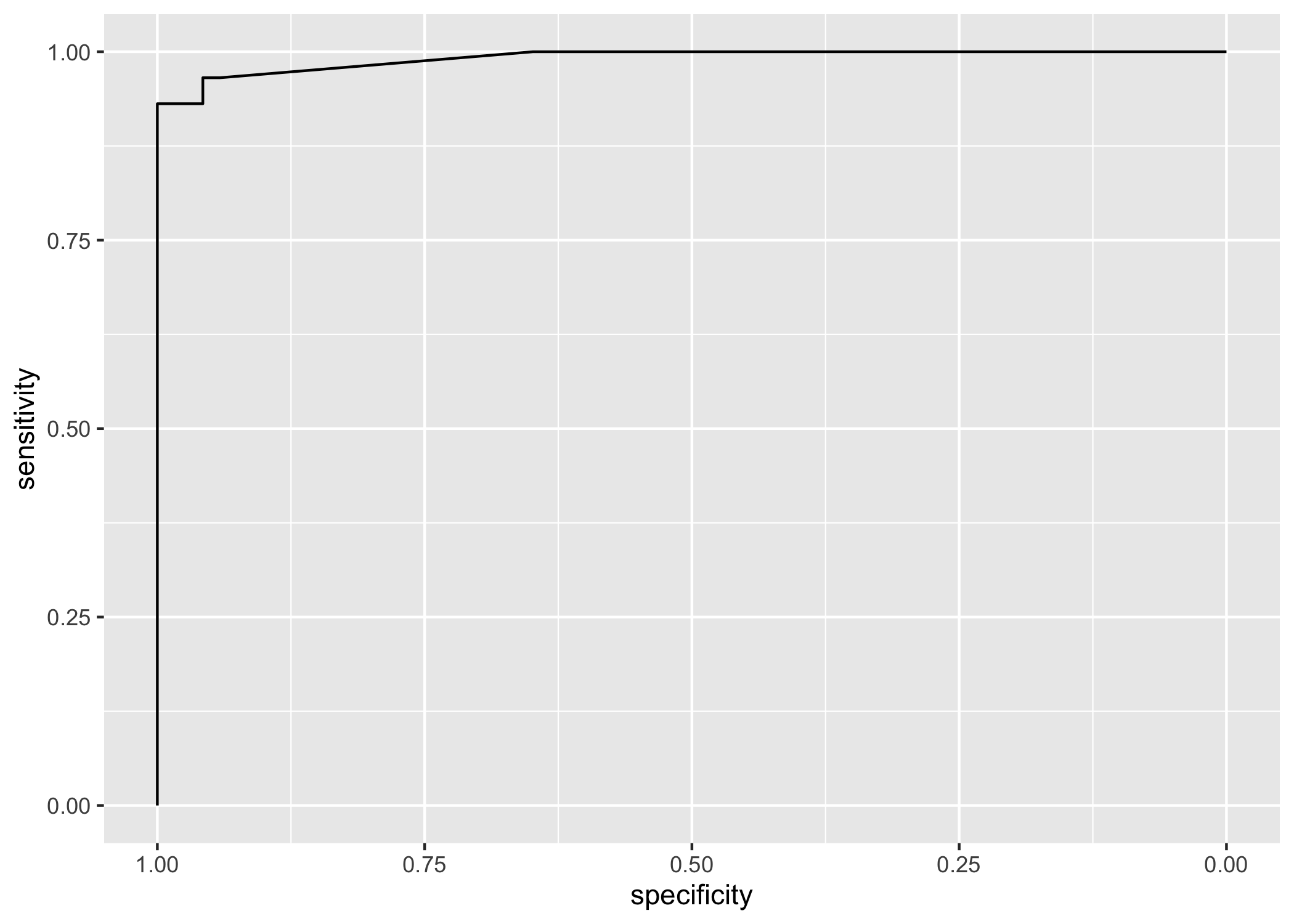
*

*Figure S1: Receiver operating characteristic curve assessing the predictive performance of the model built from equation1.*
